# EGGS: Empirical Genotype Generalizer for Samples

**DOI:** 10.1101/2025.10.16.682896

**Published:** 2025-10-17

**Authors:** T. Quinn Smith, Amatur Rahman, Zachary A. Szpiech

**Affiliations:** 1 Department of Biology, The Pennsylvania State University, University Park, PA 16802

## Abstract

We introduce Empirical Genotype Generalizer for Samples (EGGS) which accepts empirical genotypes with missing data and replicates the underlying dispersal and distribution of missing genotypes in other replicates. In addition, EGGS can remove phase, remove polarization, simulate deamination, create pseudohaploids, and convert between VCF, *ms*-style replicates, and EIGENSTRAT.

**Availability and Implementation:**

EGGS is written in the C programming language. Precompiled executables, source code, and the manual are available at https://github.com/TQ-Smith/EGGS

## 1 Introduction

Recent advantages in both forward and backward in-time simulations have made it possible to generate synthetic genotypes under varying evolutionary assumptions and complicated demographic scenarios [1, 2, 3, 4]. Such simulations are essential for rigorously testing computational methods, and more recently, training machine learning models [5, 6]. In addition, simulations are often used to test hypotheses concerning the evolutionary histories of human and nonhuman species [6, 7].

Simulated samples are reported under idyllic conditions where each genotype is phased and, in the case of biallelic sites, each site has a known ancestral allele. Simulated genotypes are devoid of technical artifacts that introduce variant uncertainty, such as missing alleles and deamination. Empirical data are often error-prone and violate the assumptions of simulated data. Ignoring this discrepancy in data quality can potentially lead to erroneous inference and unreliable results. Robust inference in the presence of low-quality genotypes is of particular interest when studying ancient DNA [8]. This has motivated the development of methods to account for genotype uncertainty [9].

Missing genotypes are caused by compounding sources, including sample quality, sequencing technology, and data processing. The inherent stochasticity and complexity of such error are randomly introduced into simulated genotypes [10]. This approach assumes that the proportion of missing samples at each site viewed in aggregate reflects the underlying structure of missingness. However, a cumulative summary of missingness ignores the dispersal of missingness across the empirical segment, which is known to be uneven in reality, especially in regions of low complexity. In addition, the random modeling of missing data using a predefined distribution may inaccurately capture the empirical distribution. These difficulties are often exacerbated in non-model organisms lacking a high-quality reference genome [11]. Several recent studies introduce missingness blocks that correspond to regions of low mappability and variant callability [12, 13]. Other current methods that introduce missingness ignore the position of the missingness, are restricted to specific simulation frameworks, and are not generalizable to any supplied VCF [14].

We introduce Empirical Genotype Generalizer for Samples (EGGS) which extracts the underlying distribution and dispersal of missing sites across a genomic region and introduces similar patterns of missingness in replicates. EGGS works for any number of sites and number of samples. We demonstrate the use of EGGS and illustrate its ability to capture realistic patterns of missing genotypes.

## 2 Materials and Methods

### 2.1 Replicating Dispersal of Missingness

Suppose we are given N sites such that the proportion of samples with both genotypes missing at site 1 ≤ *i* ≤ *N* is *x_i_*. We wish to replicate the dispersal of missingness summarized by *x* across *M* loci. Let *y_j_* for 1 ≤ *j* ≤ *M* be the result. We consider two cases: *M* ≤ *N* and *M* > *N*. In the first case, we partition *x* into blocks of $\left\lceil \frac{N}{M}\right\rceil$ loci. The last block could contain fewer loci. We assign *y_j_*, for 1 ≤ *j* ≤ *M*, by randomly selecting a proportion from the *j^th^* block of *x*. In the second case, when *M* > *N*, we partition *y* into blocks of $\left\lceil \frac{M}{N}\right\rceil$ loci. Let *U*(*a, b*) be the uniform distribution between [*a, b*). For the first 1 ≤ *j* ≤ *N*−1 blocks of *y*, we fill the block of *y* from *U*(*min*(*x_j_*, *x*_*j*+1_), *max*(*x_j_*, *x*_*j*+1_)). For the last block, we choose from *U*(*min*(*x*_*N*−1_, *x_N_*), *max*(*x*_*N*−1_, *x_N_*)). This implicitly assumes that the missingness pattern at the end of *x* can be extended.

### 2.2 Implementation

EGGS accepts variants in Variant Call Format (VCF) [15] or *ms*-style formatting [16]. In addition, we provide an option for the conversion from EIGENSTRAT format, the format used by The Allen Ancient DNA Resource (AADR), to VCF [17, 18]. In the case of multiple *ms*-style replicates as input, replicates are split to separate files VCF files. EGGS assumes all samples are diploid.

To remove phase, EGGS swaps the left and right genotype with equal probability. To remove polarization from biallelic sites, EGGS swaps the ancestral and derived alleles with equal probability. EGGS can introduce missing genotypes randomly according to the approach of Pandey et al. [10]. The proportion of samples with both alleles missing is calculated per site. The mean and standard deviation of the proportion of missing genotypes is calculated over all sites and is used to parameterize a beta-distribution. The beta-distribution is used to randomly choose the proportion of missing samples at each site in the supplied genotypes. The user can choose to directly give the mean and standard deviation to parameterize the beta-distribution or a VCF from which the values are calculated.

Deamination can be introduced according to Harney et al. [19]. The user supplies the probability that a site is a transition, and the probability that the transition will deaminate to the alternative allele if the sample originally holds the reference allele. Pseudohaploids are created by randomly choosing each sample to be homozygous for either the reference or alternative allele at biallelic sites with equal probability. Finally, summary statistics regarding missing genotypes can be calculated similar to those implemented in PLINK [20]. EGGS can produce *ms*-style output when missing data is not introduced. EGGS is written in the C programming language to enable fast processing of thousands of replicates. Precompiled executables, source code, and manual are available at https://github.com/TQ-Smith/EGGS.

## 3 Results

We used EGGS to convert the AARD [17] to VCF, and we extracted the autosomes of the 217 samples exclusively from Mathieson et al. [21] with bcftools [22]. This resulted in 1150639 VCF records. The sites had a mean missingness of 0.56 and a standard deviation of 0.21. We refer to this set as the ancient samples. Using *msprime* [2], we simulated a neutrally evolving 10 Mb region for 200 diploid samples with *Ne* = 10, 000, *μ* = 1.29 × 10^−8^, and *ρ* = 1 × 10^−8^. The simulated segment contained 33766 segregating sites. We introduced missing genotypes into the simulated segment randomly according to Pandey et al. [10] using a beta-distribution and with EGGS’s method.

**Figure 1:**
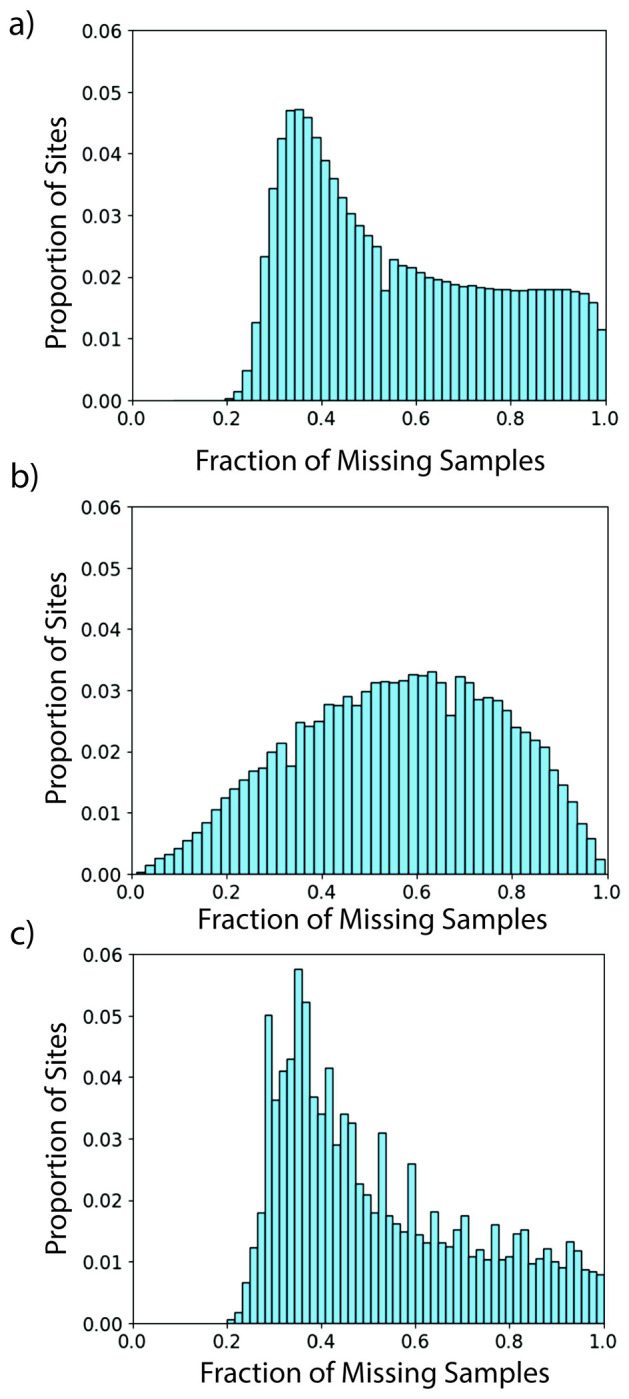
Distribution of Missingness. Each histogram was created using 50 bins. **Panel a)** The ancient samples. **Panel b)** Simulated missingness with the beta-distribution. **Panel c)** Simulated missingness with EGGS’s method.

We calculated the proportion of sites with the fraction of samples with missing genotypes for the set of ancient samples (Figure 1a), for the simulated missingness using the beta-distribution (Figure 1b), and for the simulated missingness using EGGS’s method (Figure 1c). Comparing Figure 1a and Figure 1b, we notice that Figure 1b does not possess a similar shape to that seen in Figure 1a. In addition, Figure 1b introduces sites with missingness proportions not observed in Figure 1a. Comparing Figure 1a to Figure 1c, we see that Figure 1c preserves the domain of missingness proportions and overall shape.

The Kullback–Leibler (KL) divergence measures the difference between two distributions [23]. We used the KL divergence to quantify the observed differences in Figure 1. The KL divergence between Figure 1a and Figure 1b is 13.81, and the KL divergence between Figure 1a and Figure 1c is 9.11. This supports our interpretation that the distribution of missingness created by EGGS is closer to the missingness in the ancient samples than the missingness created by the beta distribution.

## 4 Discussion

We introduced Empirical Genotype Generalizer for Samples (EGGS) that artificially removes genotypes in patterns similar to empirical data as opposed to treating the distribution and dispersal of missing data across a segment as purely random. Combining EGGS’s ability to remove genotypes and other evolutionary assumptions, we can quickly make simulated genotypes more realistic, enabling more robust downstream analyses.

EGGS’s method to replicate missingness patterns becomes limited when there is a large difference in the number of loci between the set of variation containing the patterns to replicate and the set of variation in which missing genotypes will be introduced. Such a discrepancy between the number of loci causes the resolution of EGGS’s stratification method to lose resolution. In addition, we expect that in situations where the rate of missing genotypes is high and region agnostic, EGGS’s approach will not yield significantly different than modeling missingness with a beta-distribution. Our approach could be replaced with more sophisticated compression and interpolation methods [23]. These methods can be tuned to preserve specific patterns of missing genotypes that trend along a segment. Finally, we note that our approach could be adapted to replicating other characteristics, such as homozygosity and ascertainment.

## 5 Acknowledgments

The authors would like to thank Abigail N. Sequeira and Ana V Leon-Apodaca for helpful suggestions. Computations for this research were performed using the Pennsylvania State University’s Institute for Computational Data Sciences’ Roar supercomputer. This work was supported by the National Institute of General Medical Sciences of the National Institutes of Health award number R35GM146926 (ZAS).

## References

[1] Haller Benjamin C, Ralph Peter L, and Messer Philipp W. Slim 5: Eco-evolutionary simulations across multiple chromosomes and full genomes. bioRxiv, pages 2025–08, 2025. 10.1093/molbev/msaf313 PMC12759289 41292177

[2] Kelleher Jerome, Etheridge Alison M, and McVean Gilean. Efficient coalescent simulation and genealogical analysis for large sample sizes. PLoS computational biology, 12(5):e1004842, 2016. 27145223 10.1371/journal.pcbi.1004842 PMC4856371

[3] Lauterbur M Elise, Cavassim Maria Izabel A, Gladstein Ariella L, Gower Graham, Pope Nathaniel S, Tsambos Georgia, Adrion Jeffrey, Belsare Saurabh, Biddanda Arjun, Caudill Victoria, Expanding the stdpopsim species catalog, and lessons learned for realistic genome simulations. Elife, 12:RP84874, 2023. 37342968 10.7554/eLife.84874 PMC10328510

[4] Kern Andrew D and Schrider Daniel R. Discoal: flexible coalescent simulations with selection. Bioinformatics, 32(24):3839–3841, 2016. 27559153 10.1093/bioinformatics/btw556 PMC5167068

[5] Rahman Amatur, Smith Thomas Quinn, and Szpiech Zachary A. Fast and memory-efficient dynamic programming approach for large-scale ehh-based selection scans. bioRxiv, pages 2025–04, 2025. 10.1093/molbev/msaf275 PMC12659807 41140120

[6] Amin Md Ruhul, Hasan Mahmudul, and DeGiorgio Michael. Digital image processing to detect adaptive evolution. Molecular Biology and Evolution, 41(12):msae242, 2024. 39565932 10.1093/molbev/msae242 PMC11631197

[7] Rayner Jack G, Eichenberger Franca, Bainbridge Jessica VA, Zhang Shangzhe, Zhang Xiao, Yusuf Leeban H, Balenger Susan, Gaggiotti Oscar E, and Bailey Nathan W. Competing adaptations maintain nonadaptive variation in a wild cricket population. Proceedings of the National Academy of Sciences, 121(32):e2317879121, 2024. 10.1073/pnas.2317879121 PMC11317585 39088392

[8] Williams Matthew P and Huber Christian D. The genomic footprints of migration: how ancient dna reveals our history of mobility. Genome Biology, 26(1):1–29, 2025. 40671036 10.1186/s13059-025-03664-w PMC12265385

[9] Bailey Nick, Stevison Laurie, and Samuk Kieran. Correcting for bias in estimates of *θ* w *θ* _w and tajima’s d d from missing data in next-generation sequencing. Molecular Ecology Resources, page e14104, 2025. 40125978 10.1111/1755-0998.14104 PMC12225706

[10] Pandey Devansh, Harris Mariana, Garud Nandita R, and Narasimhan Vagheesh M. Leveraging ancient dna to uncover signals of natural selection in europe lost due to admixture or drift. Nature Communications, 15(1):9772, 2024. 10.1038/s41467-024-53852-8 PMC11557891 39532856

[11] Mora-Márquez Fernando, Nuño Juan Carlos, Soto Álvaro, and de Heredia Unai López. Missing genotype imputation in non-model species using self-organizing maps. Molecular Ecology Resources, 25(3):e13992, 2025. 38970328 10.1111/1755-0998.13992 PMC11887599

[12] Arnab Sandipan Paul, dos Santos Andre Luiz Campelo, Fumagalli Matteo, and DeGiorgio Michael. Efficient detection and characterization of targets of natural selection using transfer learning. Molecular Biology and Evolution, 42(5):msaf094, 2025. 40341942 10.1093/molbev/msaf094 PMC12062966

[13] Skov Laurits, Hui Ruoyun, Shchur Vladimir, Hobolth Asger, Scally Aylwyn, Schierup Mikkel Heide, and Durbin Richard. Detecting archaic introgression using an unadmixed outgroup. PLoS genetics, 14(9):e1007641, 2018. 30226838 10.1371/journal.pgen.1007641 PMC6161914

[14] Goulart Paimon and Samuk Kieran. vcfsim: flexible simulation of all-sites vcfs with missing data. bioRxiv, pages 2025–01, 2025. 10.1186/s12859-026-06453-9 PMC13267514 42050398

[15] Danecek Petr, Auton Adam, Abecasis Goncalo, Albers Cornelis A, Banks Eric, DePristo Mark A, Handsaker Robert E, Lunter Gerton, Marth Gabor T, Sherry Stephen T, The variant call format and vcftools. Bioinformatics, 27(15):2156–2158, 2011. 21653522 10.1093/bioinformatics/btr330 PMC3137218

[16] Hudson Richard R. Generating samples under a wright–fisher neutral model of genetic variation. Bioinformatics, 18(2):337–338, 2002. 11847089 10.1093/bioinformatics/18.2.337

[17] Mallick Swapan and Reich David. The Allen Ancient DNA Resource (AADR): A curated compendium of ancient human genomes, 2023. URL 10.7910/DVN/FFIDCW. PMC10858950 38341426

[18] Mallick Swapan, Micco Adam, Mah Matthew, Ringbauer Harald, Lazaridis Iosif, Olalde Iñigo, Patterson Nick, and Reich David. The allen ancient dna resource (aadr) a curated compendium of ancient human genomes. Scientific Data, 11(1):182, 2024. 38341426 10.1038/s41597-024-03031-7 PMC10858950

[19] Harney Éadaoin, Patterson Nick, Reich David, and Wakeley John. Assessing the performance of qpadm: a statistical tool for studying population admixture. Genetics, 217(4):iyaa045, 2021. 33772284 10.1093/genetics/iyaa045 PMC8049561

[20] Purcell Shaun, Neale Benjamin, Todd-Brown Kathe, Thomas Lori, Ferreira Manuel AR, Bender David, Maller Julian, Sklar Pamela, De Bakker Paul IW, Daly Mark J, Plink: a tool set for whole-genome association and population-based linkage analyses. The American journal of human genetics, 81(3):559–575, 2007. 17701901 10.1086/519795 PMC1950838

[21] Mathieson Iain, Alpaslan-Roodenberg Songül, Posth Cosimo, Szécsényi-Nagy Anna, Rohland Nadin, Mallick Swapan, Olalde Iñigo, Broomandkhoshbacht Nasreen, Candilio Francesca, Cheronet Olivia, The genomic history of southeastern europe. Nature, 555(7695):197–203, 2018. 29466330 10.1038/nature25778 PMC6091220

[22] Danecek Petr, Bonfield James K, Liddle Jennifer, Marshall John, Ohan Valeriu, Pollard Martin O, Whitwham Andrew, Keane Thomas, McCarthy Shane A, Davies Robert M, Twelve years of samtools and bcftools. Gigascience, 10(2):giab008, 2021. 33590861 10.1093/gigascience/giab008 PMC7931819

[23] Sayood Khalid. Introduction to data compression. Morgan Kaufmann, 2017.

